# Ratiometric G-Quadruplex Assay for Robust Lead Detection in Food Samples

**DOI:** 10.3390/bios11080274

**Published:** 2021-08-16

**Authors:** Yumei Liu, Hao Yang, Rui Wan, Mohammad Rizwan Khan, Nan Wang, Rosa Busquets, Ruijie Deng, Qiang He, Zhifeng Zhao

**Affiliations:** 1 College of Biomass Science and Engineering, Healthy Food Evaluation Research Center and Key Laboratory of Food Science and Technology of Ministry of Education of Sichuan Province, Sichuan University, Chengdu 610065, China; liuyumei@stu.scu.edu.cn (Y.L.); hyang_2018@163.com (H.Y.); wr2339077579@163.com (R.W.); thanksmyfriends@163.com (N.W.); drj17@scu.edu.cn (R.D.); heq361@163.com (Q.H.); 2 Department of Chemistry, College of Science, King Saud University, Riyadh 11451, Saudi Arabia; mrkhan@KSU.EDU.SA; 3 School of Life Sciences, Pharmacy and Chemistry, Kingston University London, Penrhyn Road, Kingston-upon-Thames, Surrey KT1 2EE, UK; r.busquets@kingston.ac.uk

**Keywords:** G-quadruplex, nucleic acid probes, lead pollution, food safety, homogeneous detection

## Abstract

Lead (Pb^2+^) pollution is a serious food safety issue, rapid detection of Pb^2+^ residual in food is vital to guarantee food quality and safety. Here we proposed ratiometric aptamer probes, allowing robust Pb^2+^ supervision in food samples. Pb^2+^ specific aptamer can bolster a transition of G-quadruplex structural response to Pb^2+^; this process can be monitored by N-methyl mesoporphyrin IX (NMM), which is highly specific to G-quadruplex. Particularly, the utilization of G-quadruplex specific dye and terminal-labeled fluorophore allowed to endue ratiometric signal outputs towards Pb^2+^, dramatically increase the robustness for lead detection. The ratiometric G-quadruplex assay allowed a facile and one-pot Pb^2+^ detection at room temperature using a single-stranded DNA aptamer. We demonstrated its feasibility for detecting lead pollution in fresh eggs and tap water samples. The ratiometric G-quadruplex design is expected to be used for on-site Pb^2+^ testing associated with food safety.

## 1. Introduction

Lead (Pb^2+^) pollution has received worldwide attention for its non-biodegradability [1], long half-life [2], bioaccumulation [3], high mobility [4,5], and high health risks to humans [6]. Since soil is a harder-hit area of Pb^2+^ pollution [7,8], Pb^2+^ is ubiquitous in raw-food materials such as rice, apples, eggs, grapes, corn, and tomatoes [9,10,11,12,13] due to bioaccumulation. Although Pb^2+^ concentration in foods is relatively low, bioaccumulation concentrates it through the food chain and further threatens people’s health [14]. Besides diet, exposure pathways toward Pb^2+^ are diversified; inhalation [15], absorption through the skin [16], and contact with Pb^2+^ polluted soils and dust are also potential pollution sources. Long time exposure to Pb^2+^ results in various diseases, such as cancer, genotoxicity, and neurological disorders [17,18,19]. Tools for the rapid detection of lead pollution are in high demand.

Standard methods based on sophisticated instruments and complicated operations have been established to accurately quantify Pb^2+^, such as atomic absorption spectrometry (AAS) [20], mass spectra (MS) [21], X-ray fluorescence spectrometry [22], and laser ablation inductively coupled plasma mass spectrometry (LA-ICP-MS) [23,24]. Though serving as gold standards for Pb^2+^ quantification, conventional methods demand professional staff, ponderous instruments, and complicated procedures, which might be unsuitable for practical application in developing countries and remote areas [25]. Thus, there is an urgent demand for facile and on-site Pb^2+^ detection technologies.

Until now, many methods related to G-quadruplex aptamer were designed for Pb^2+^ detection [26,27,28,29,30]. The sensing mechanism was the conformation switching of the G-quadruplex itself. G-quadruplex is a non-classical secondary nucleic acid structure [31]. The addition of metal ions can improve the stability of the G-quadruplex, and potassium ions (K^+^) can trigger the formation of parallel G-quadruplex configurations [32]. Porphyrins, such as NMM, could specifically bind with G-quadruplex and enhance their fluorescence upon binding [33]. Thus, NMM could be used as a fluorescent indicator for the presence of G-quadruplex. In addition to Pb^2+^, the parallel G-quadruplex was induced to form an unparallel structure [34]; the cavity within the G-quadruplex became smaller and could no longer bind with NMM, so the fluorescence of NMM would be significantly reduced. Thus, NMM could be used to monitor the presence of Pb^2+^. These single-signal outputs methods, however, suffer from drawbacks arising from the fluctuation of probe concentrations [35], as well as the instrumental variations.

**Figure 1:**
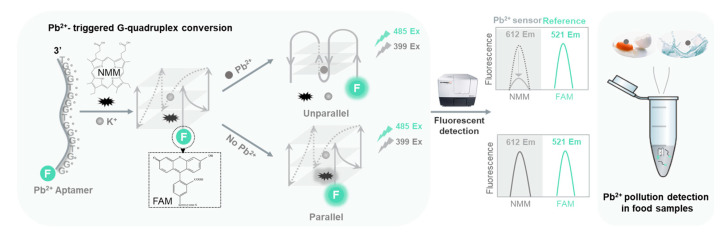
Working principle of the aptasensor and its application for Pb^2+^ pollution detection. NMM refers to N-methyl Mesoporphyrin IX, and FAM refers to 6-carboxy-fluorescein.

The programmability of DNA sequences allows for constructing synthetic molecular sensors [36]. Here, a ratiometric fluorescent strategy was proposed for robust and sensitive Pb^2+^ detection in food samples. G-quadruplex specific dye, NMM, was used for sensing the presence of Pb^2^^+^; chemically labeled 6-carboxy-fluorescein (FAM) was used as a reference, sequentially, and contributed to a ratiometric Pb^2+^ assay. The design principle is illustrated in Figure 1. K^+^ has the feature of stabilizing G-quadruplex [37]; upon the addition of K^+^, the linear aptamer naturally folds into a parallel structure with the incorporation of K^+^, thus allowing the binding and turning on of NMM. The presence of Pb^2+^ rapidly turns the aptamer into an unparallel G-quadruplex structure, which frees NMM dye, and sharply reduced the fluorescence of NMM. Thus, the presence of Pb^2+^ can be monitored via the fluorescence measurement of NMM. In contrast, the terminal labeled FAM emits a fluorescence that is not affected by G-quadruplex conversion, thus serving as a reference for detecting Pb^2+^. We showed that the ratiometric detection strategy could resist the variation of probe concentrations, thus dramatically improving the assaying robustness. We further demonstrated its use for detecting lead pollution in water and food samples. The ratiometric fluorescent aptasensor allows one-pot Pb^2+^ detection at room temperature, thus promising on-site monitoring of lead pollution associated with food safety.

## 2. Materials and Methods

### 2.1. Materials and Instrumentations

The G-quadruplex probe for Pb^2+^ detection was 5′-GGGTGGGTGGGTGGGT-3′ [38]. It was chemically labeled with a FAM at its 5′-terminal, which was HPLC purified. Tris-HCl and labeled aptamer were obtained from Shanghai Sangon Biological Engineering Technology & Services Co., Ltd. (Shanghai, China). Pb (CH_3_COO)_2_ was purchased from Sigma Aldrich (Mississauga, ON, Canada). Mn (CH_3_COO)_2_ was purchased from Tianjin Damao Chemical Reagent Factory (Tianjin, China), CoCl_2_, Cd (NO_3_)_2_, CuSO_4_, Al (NO_3_)_3_, KCl, NiCl_2_, MgCl_2_, HNO_3_, and HClO_4_ were purchased from Chengdu Kelon Chemical Reagent Factory (Chengdu, China). HgCl_2_ was purchased from Tongren Chemical Industry Research Institute (Tongren, China). NMM was provided by Santa Cruz Biotechnology, Inc. (Dallas, TX, USA). Molecular biology-grade water was provided by Corning (Corning, NY, USA) and used for solution preparation.

### 2.2. Pb^2+^ Detection

For the Pb^2+^ detection procedures, the mixture was composed of 4 μL 10× buffer (330 mM Tris-HCl, 660 mM KCl, 100 mM MgCl_2_, pH 8.0 at 25 °C), 4 μL FAM-labeled aptamer (5 μM), 4 μL NMM (20 μM), 4 μL Pb^2+^ solution in different concentrations and 24 μL H_2_O were added to make a total of 40 μL capacity. 35 μL of the mixture was taken to read the fluorescent intensity with microplate reader Synergy H1 after 30 min incubation at room temperature.

### 2.3. Fluorescent Analysis Method

NMM was excited at 399 nm and measured every 2 nm (550 nm to 700 nm was the emission wavelength in range). For reference dye FAM, it was excited at 485 nm and measured every 2 nm (515 nm to 699 nm was the emission wavelength in range). Real-time fluorescent intensity of NMM and FAM was collected within 1 h on the microplate reader Synergy H1 (Vermont, USA). For NMM, it was excited at 399 nm (collected at 612 nm) and measured every 60 s. For FAM, it was excited at 485 nm (collected at 521 nm) and measured every 60 s.

### 2.4. Selectivity Test for Pb^2+^ Detection

Heavy metal contamination is a common problem for food safety control [39]. Lead contamination in food is always accompanied by other heavy metal contamination such as Cu^2+^, Co^2+^, Cd^2+^, Mn^2+^, Al^3+^, Hg^2+^, and Ni^2+^. To verify the specificity of our proposed method, 100 nM, 300 nM, and 700 nM of metal ions were added to the reaction system for detection, respectively. All the testing operations were consistent with what was mentioned above. All tests were repeated three times in parallel.

### 2.5. Food Samples Detection

Spiked recovery assay was used to determine the application potential of the established method. Fresh eggs and tap water were used to test the feasibility. Fresh eggs were obtained from a local supermarket (Chengdu, China). Tap water was obtained from the laboratory. Before spiking different concentrations of Pb^2+^, fresh egg liquid was mixed well for digestion. In addition to 10 mL HNO_3_, 0.5 mL well-mixed egg liquid, 0.5 mL HClO_4_ were added, and the digestion tube was placed in the digestive furnace and programmed as 120 °C for 1 h, 180 °C for 3 h, 200 °C for 1 h. After that, 1 M KOH was added to adjust the pH of the remaining liquid to about pH 7.0. The digestive experiments mentioned above were based on the Chinese National Standard for Food Safety—Determination of Lead Content in Food Products (GB 5009.12-2017, in Chinese) with minor adjustments. Different amounts of Pb^2+^ were artificially mixed with the prepared liquid. Finally, the prepared liquid was subjected to further analysis with the procedures mentioned above. Recovery rate was used to reflect the precision and feasibility of the established method, and it was calculated with the following formula:

$$P\;=\;\frac{C_{1}-C_{2}}{C_{0}}\;\times 100\%$$

where *P* represented the recovery rate, *C*_1_ represented the concentration of Pb^2+^ found in the food samples spiking different concentrations of Pb^2+^, which was calculated with the linear regression equation by introducing the fluorescence collected. *C*_2_ represented the concentration of Pb^2+^ found in the food samples without adding Pb^2+^. *C*_0_ represented the concentration of Pb^2+^ artificially added to the samples, which was directly decided by the linear range. A relatively lower, medium, higher Pb^2+^ amount within the linear range was spiked into the samples to validate the feasibility of the method. The experimental conditions and operations were the same as the linear regression equation.

## 3. Results and Discussion

### 3.1. Principle and Robustness of the Assay

**Figure 2:**
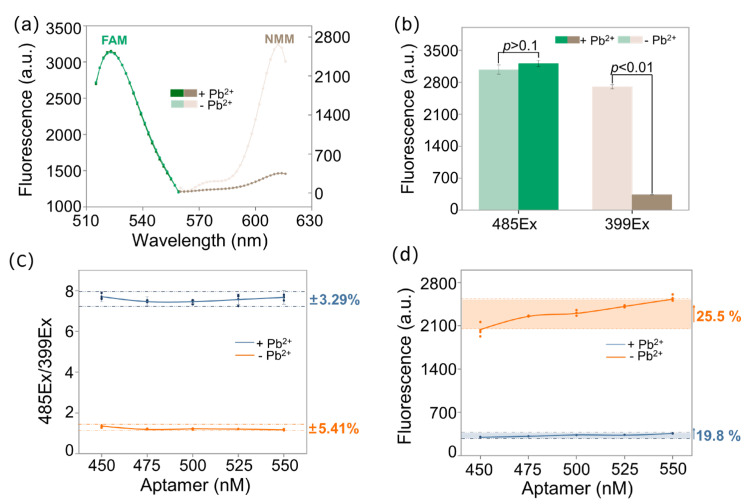
Principle verification and robustness of the ratiometric assay. (**a**) Fluorescent spectra of the aptasensor. (**b**) Fluorescent intensity histogram in the presence and absence of Pb^2+^, respectively. (**c**) Robustness of the G-quadruplex probes. 450, 475, 500, 525, and 550 nM aptamer were tried, respectively. 485Ex/399Ex was the ratio of fluorescent intensity collected at 521 nm (485 nm excitation) to fluorescent intensity collected at 612 nm (399 nm excitation). (**d**) Corresponding fluorescent intensity of (**c**).

As shown in Figure 2a,b, the fluorescent intensity of FAM (both the green and light green lines and the green and light green columns) was not dramatically changed after adding Pb^2+^, which showed Pb^2+^, as well as Pb^2+^-triggered G-quadruplex conversion, did not influence the fluorescence of FAM significantly. Thus, FAM had the potential to serve as a reference dye for detecting Pb^2+^. For NMM (both the brown and light brown lines and the brown and light brown columns), the fluorescent intensity decreased obviously in the presence of Pb^2+^, which hinted that Pb^2+^ could displace K^+^ and NMM as soon as possible and it has higher efficiency stabilizing G-quadruplex compared with K^+^. Thus, the fluorescent change of NMM could be used to indicate the presence of Pb^2+^ and monitor lead pollution.

Different concentrations of aptamer (450, 475, 500, 525, and 550 nM) were added to the detection system to determine if the proposed ratiometric assay was capable of relieving the fluctuation caused by varied aptamer concentrations. As shown in Figure 2c, when the amounts of aptamer were in the range of 450 to 550 nM, 485Ex/399Ex fluctuated in a small range. The fluctuation range in the absence of Pb^2^^+^ (the orange line) was within ±5.41%, and the fluctuation range in the presence of Pb^2+^ (the blue line) was within ±3.29%. As shown in Figure 2d, when the aptamer concentrations varied from 450 to 550 nM, for the background (the orange line), the fluorescent intensity increased by 25.5% with the increase of the aptamer; on the contrary, the fluorescent intensity in the presence of Pb^2+^ increased by 19.8%. Thus, the chemically labeled FAM was able to reduce the signal fluctuation caused by varied aptamer concentrations, so the robustness of the proposed assay was verified.

### 3.2. Optimization of the Experimental Conditions

**Figure 3:**
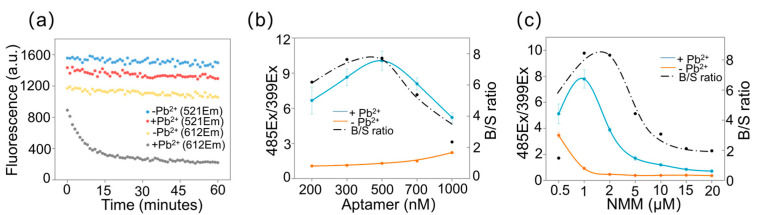
(**a**) Real-time fluorescence of FAM (excited at 485 nm and collected at 521 nm) in the presence (the red dotted line) and absence (the blue dotted line) of Pb^2+^ and real-time fluorescence of NMM (excited at 399 nm and collected at 612 nm) in the presence (the grey dotted line) and absence (the yellow dotted line) of Pb^2+^. (**b**) Optimization of aptamer concentrations (200, 300, 500, 700, 1000 nM). (**c**) Optimization of NMM concentrations (0.5, 1, 2, 5, 10, 15 and 20 μM).

To get better results, experimental conditions including incubation time, aptamer concentration, and NMM concentration were optimized, respectively (Figure 3). The fluorescence of NMM without Pb^2+^ (the yellow dot line) changed slightly as time went by. The real-time fluorescent intensity of NMM with the addition of Pb^2+^ (the light grey dotted line) indicated that the response of NMM dye towards the presence of Pb^2+^ was finished within 30 min (Figure 3a). What is more, the slightly dropped fluorescence of FAM with Pb^2+^ (the red dotted line) may be due to photobleaching. Thus, the incubation time was set as 30 min. Next, aptamer concentration was optimized. The signal of 485Ex/399Ex rose with the increase of aptamer and maximized when the aptamer concentration was 500 nM (Figure 3b). Moreover, the background to signal (B/S) ratio, which could suggest the sensitivity of our assay, was also maximized when the concentration of aptamer was 500 nM. So, 500 nM of aptamer was the optimized concentration of the aptamer probe. Since NMM directly supervised the conversion process from parallel G-quadruplex to unparallel G-quadruplex, so, the concentration of NMM was optimized next. As shown in Figure 3c, the B/S peaked when the concentration of NMM was 2 μM. Thus, 2 μM NMM was used in the following experiments:

### 3.3. Quantification Performance of Pb^2+^ Assay

**Figure 4:**
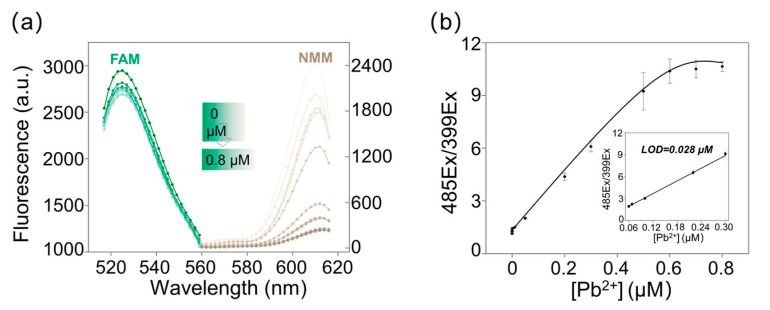
Quantitative experiment of Pb^2+^ with the proposed assay. (**a**) Fluorescent spectrum collected at 521 nm (FAM, excited at 425 nm) and collected at 612 nm (NMM, excited at 399 nm) respectively. 0, 0.0001, 0.001, 0.01, 0.05, 0.2, 0.3, 0.5, 0.6, 0.7 and 0.8 μM Pb^2^^+^ were added, respectively. (**b**) The relationship between Pb^2+^ concentration and 485Ex/399Ex; (Insert) 485Ex/399E showed a linear relationship with the increase of Pb^2+^.

To assess the sensitivity of the proposed ratiometric assay, different concentrations of Pb^2+^ (0–0.8 μM) were added under the optimized conditions, as illustrated in Figure 4a, for FAM, the fluorescent intensity negligibly decreased with the increase of Pb^2+^, which demonstrated FAM could work as a reference dye. Meanwhile, the fluorescent intensity of NMM obviously decreased with the increase of Pb^2+^, which indicated that the fluorescence of NMM was Pb^2+^-dependent and the fluorescence of FAM was Pb^2+^-independent. Thus, it was possible to establish a ratiometric G-quadruplex strategy that might reduce the fluctuation caused by varied probes concentration. A linear relationship between different concentrations of Pb^2+^ and 485Ex/399Ex was calculated, therefore. As shown in Figure 4b, the ratio 485Ex/399Ex increased linearly with the increase of Pb^2+^ (0.06 μM to 0.3 μM). The linear relationship between the 485Ex/399Ex ratio and Pb^2+^ concentration was calculated as y = 16.67 x + 1.31 (R^2^ = 0.993), where x and y stood for Pb^2+^ concentration and 485Ex/399Ex, respectively. The limit of detection (LOD) of the ratiometrix G-quadruplex aptasensor was 0.028 μM (5.89 ppb) according to 3б rule. This indicated that our design was more sensitive than Wu’s work, whose LOD was 58.59 nM. For the comparison with Wu’s work [32], DAPI was used as a reference channel, and the key design was the dual fluorescence emission at 610 nm and 450 nm. Since not all the DAPI would be successfully and totally trapped in the parallel or unparallel G-quadruplex (due to the G-quadruplex conversion), it is hard to find the appropriate concentration of DAPI to characterize the presence of G-quadruplex, either parallel ones or unparallel ones. So, in our work, we chemically labeled FAM at the 5′terminal of the aptamer, the fluorescence of FAM was much more controllable by adding a specific amount of aptamer. The labeled FAM would make the experimental results more precise and repeatable. The design of our work was rather simple, and NMM was the only dye needed to supervise the conversion of G-quadruplex, which is commercially available and cheap. Comparations among DNAzyme-based biosensors for lead detection could be found in Table S2 in the Supplementary Materials.

### 3.4. Specific Test

**Figure 5:**
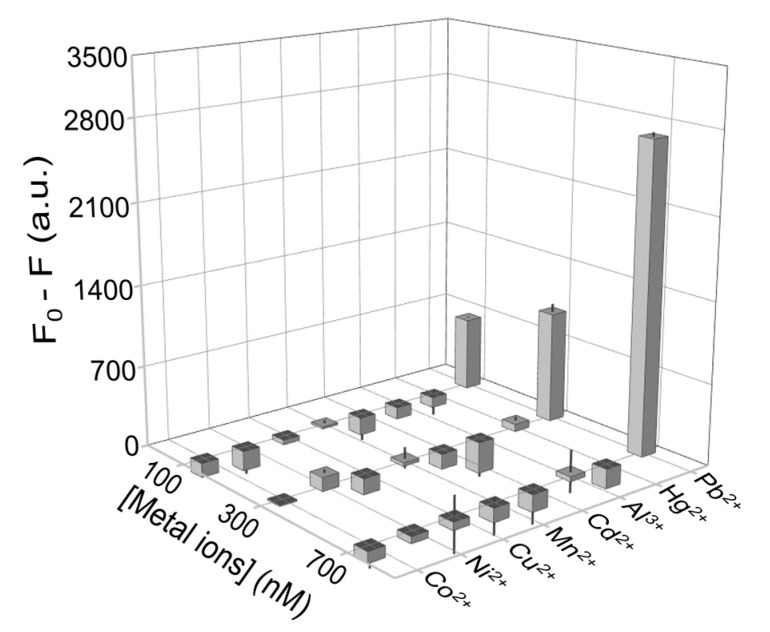
Corresponding signal response of the aptasensor towards different concentrations of metal ions (Pb^2+^, Cd^2+^, Co^2+^, Al^3+^, Mn^2+^, Ni^2+^, Cu^2+^, Hg^2+^) at the concentration of 100, 300 and 700 nM.

Due to biological accumulation and non-biodegradable features of Pb^2+^, lead contamination in food is always accompanied by other heavy metal ions, such as Cd^2+^, Mn^2+^, Cu^2+^, Co^2+^, Al^3+^, Hg^2+^ and Ni^2+^ [40,41,42,43,44,45,46]. Thus, it is of vital importance to avoid their disturbance with Pb^2+^ detection. 100, 300, and 700 nM of various metal ions were spiked into the mixture for detection. F_0_ represented the fluorescence of NMM without adding metal ions, F represents the fluorescence of NMM after adding different concentrations of metal ions. The corresponding F_0_-F of the aptamer to Cd^2+^, Co^2+^, Al^3+^, Mn^2+^, Ni^2+^, Cu^2+^, and Hg^2+^, were all around zero while the F_0_-Fwere around 600, 800, and 2400 when the concentration of Pb^2+^ were 100, 300, and 700 nM respectively (see Figure 5 and Figure S1 in the Supplementary Materials). What’s more, according to Du’s work [47], the formation of T-Hg-T could hinder the proper folding of the G-quadruplex structure and inhibit the activity of DNAzyme. In our work, however, the fluorescence of NMM in the presence of Hg^2^^+^ was the same as other metal ions, which meant G-quadruplex was formed, and NMM was trapped inside. We reason that T in (G_3_T)_4_ was not enough in the detection system to form enough T-Hg-T to hinder the K^+^-induced G-quadruplex. This indicated that within the linear range, the ratiometric fluorescent aptasensor was sensitive enough to detect Pb^2+^ even in the presence of Hg^2+^ Thus, the proposed ratiometric fluorescent aptasensor could be a potential toolkit to recognize Pb^2+^ in a real food sample.

### 3.5. Detection of Lead Pollution in Food Samples

**Figure 6:**
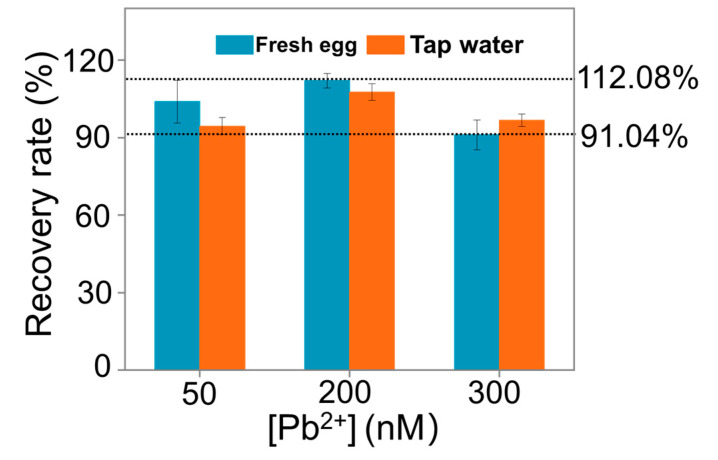
Application of the ratiometric fluorescent aptasensor. 50, 200, 300 nM Pb^2+^ were spiked into the actual samples to verify the practicability of the ratiometric fluorescent aptasensor.

Food samples are complex, which may inhibit metal ions detection. In order to demonstrate the feasibility of our method, tap water and fresh eggs artificially contaminated with 50, 200, and 300 nM of Pb^2+^ were prepared for detection, respectively. As shown in Figure 6, the recovery values of Pb^2+^ in these samples ranged from 91.04% to 112.08% (see detailed information in Table S1 in the Supplementary Materials). These results confirmed the feasibility of the designed aptasensor for Pb^2+^ supervision in food samples.

## 4. Conclusions

Here, we proposed a fast and ratiometric fluorescent aptasensor for Pb^2+^ detection with the help of terminal-labeled DNA aptamers. The combined utilization of G-quadruplex specific dye NMM and terminal-labeled FAM fluorophore in the detection system dramatically improved the robustness for detecting Pb^2+^. The G-quadruplex assay could distinguish Pb^2+^ from other metal ions, enduing a high selectivity for Pb^2+^ detection. We successfully applied the assay in detecting Pb^2+^ in tap water and fresh eggs with high accuracy. Remarkably, it allowed one-pot Pb^2+^ detection at room temperature. A portable fluorometer is expected to combine with our assay for on-site monitoring of lead contamination in different complex samples, thus providing a promising point-of-care test regarding food safety.

## Data Availability

Not applicable.

## Supplementary Materials

The following are available online at https://www.mdpi.com/article/10.3390/bios11080274/s1, Figure S1: Fluorescent response of the Pb^2+^ aptamer probe towards different metal ions at concentrations of 100 nM, 300 nM and 700 nM, Table S1: Determination of Pb^2+^ spiked in fresh egg and tap water, Table S2: Comparations among DNAzyme-based biosensors for lead detection.
