# Supplementary material for: Ratiometric G-Quadruplex Assay for Robust Lead Detection in Food Samples
Source: Biosensors (Basel). 2021 Aug 16;11(8):274. doi: 10.3390/bios11080274 (PMC8391220; doi:10.3390/bios11080274)
Supplement: Supplementary file 1 [file biosensors-11-00274-s001.zip › biosensors-1285248-supplementary.pdf]

Supporting information

Ratiometric G-quadruplex Assay for Robust Detection of Lead Pollution in Food Samples

Yumei Liu[1], Hao Yang[1], Rui Wan[1], Mohammad Rizwan Khan[2], Nan Wang[1], Rosa Busquets[3], Ruijie Deng[1], Qiang He[1] and Zhifeng Zhao[1,*]

[1] College of Biomass Science and Engineering, Healthy Food Evaluation Research Center and Key Laboratory of Food Science and Technology of Ministry of Education of Sichuan Province, Sichuan University, Chengdu 610065, China; liuyumei@stu.scu.edu.cn (Y.L.); hyang_2018@163.com (H.Y.); wr2339077579@163.com (R.W.); thanksmyfriends@163.com (N.W.); drj17@scu.edu.cn (R.D.); heq361@163.com (Q.H.)

[2] Department of Chemistry, College of Science, King Saud University, Riyadh 11451, Saudi Arabia; mrkhan@KSU.EDU.SA

[3] School of Life Sciences, Pharmacy and Chemistry, Kingston University London, Penrhyn Road, KT12EE, Kingston Upon Thames, United Kingdom; r.busquets@kingston.ac.uk

* Correspondence: zhaozhifeng@scu.edu.cn; Tel.: +86-028-8546-7328

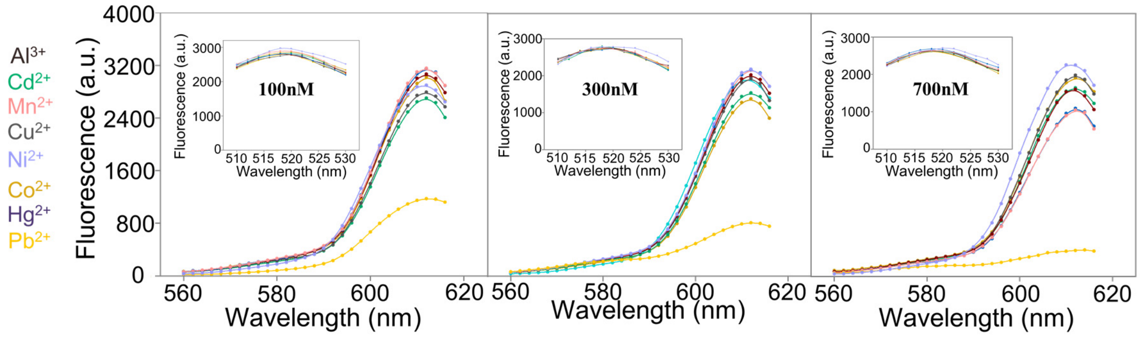

Figure S1 Fluorescent response of the Pb^{2+} aptamer probe towards different metal ions at concentrations of 100 nM, 300 nM and 700 nM.

Table S1 Determination of Pb^{2+} spiked in fresh egg and tap water

samples	Added (nM)	Found (nM)	Recovery (%)	RSD(%) n=3
Fresh egg	50	52.00	104.00	8.27
	200	224.17	112.08	2.79
	300	273.13	91.04	5.79
Tap water	50	47.23	94.47	3.30
	200	215.28	107.64	3.24
	300	290.27	96.76	2.42

Table S2. Comparations among DNAzyme-based biosensors for lead detection

Methods		LOD	Linear range	Detection time	Real sample	References
Fluorescent sensors	**1**	28 nM	60 - 300 nM	30 min	Fresh egg, tap water	*This work*
	**2**	18 nM	0 - 480 nM	25 min	Water	[1]
	**3**	0.42 nM	0 - 100 nM	30 min	Tap water, serum	[2]
	**4**	20 nM (fisrt cycle)<br>5 nM (second cycle)	20 - 1000 nM(fisrt cycle)<br>0.01 - 2μM (second cycle)	5 h	-	[3]
	**5**	10 nM	10 - 1000 nM	2 h	River water, sewage water, lake water, human body, sea water	[4]
	**6**	58.59 nM	100 - 2500 nM	10 min	Black tea, tap water	[5]
	**7**	0.6 nM	9.9 - 435.0 nM	5 min		[6]
	**8**	3.6 nM	0.1 - 1.0 μM	400 min	Human serum	[7]
	**9**	11.7 pM	0.1 - 1000 nM	30 min	Tap water, serum	[8]
	**10**	23.5 nM	100 - 600 nM	3 h 10 min	-	[9]
	**11**	SWCNHs (9.7 nM),<br>GO (10.8 nM)	0 - 0.9 μM	-	-	[10]
	**12**	8.70 pM	10.0 pM - 1000.0 nM	5 min	Tap water,fertilizer	[11]
Colorimetric sensors	**1**	0.61 nM	0 - 750 nM	2 min	-	[12]
	**2**	192 pM	0.01 – 1 nM	7 min	-	[13]
Electrochemical sensors	**1**	9 fM	1.0×10^{-14} - 1.0×10^{-11} M	3 h 30 min	River water	[14]
	**2**	0.0042 nM	0.01 - 200 nM	60 min	Water, soil	[15]
	**3**	0.6 ppb	0.5 - 25 ppb	75 min	Soil	[16]
	**4**	0.032 pM	0.1 pM - 100 nM	2 h 20 min	Tap water, lake water	[17]
	**5**	0.4 nM	1.0 nM - 1.0 μM	10 min	River water	[18]
	**6**	CPE: 1.04×10^{-10} M<br>MWCNTPE: 2.64×10^{-11} M	CPE<br>4.0×10^{-10} - 5.0×10^{-9} M<br>2.0×10^{-7} - 1.0×10^{-5} M<br>MWCNTPE<br>4.0×10^{-11} - 1.0×10^{-9} M<br>2.0×10^{-7} - 1.0×10^{-5} M	5 min	Tap water, sea water	[19]

References

1. Zhan, S.; Wu, Y.; Luo, Y.; Liu, L.; He, L.; Xing, H.; Zhou, P. Label-Free Fluorescent Sensor for Lead Ion Detection Based on Lead(II)-Stabilized G-Quadruplex Formation. *Analytical Biochemistry* **2014**, *462*, 19–25, doi:10.1016/j.ab.2014.01.010.
2. Taghdisi, S.M.; Emrani, S.S.; Tabrizian, K.; Ramezani, M.; Abnous, K.; Emrani, A.S. Ultrasensitive Detection of Lead (II) Based on Fluorescent Aptamer-Functionalized Carbon Nanotubes. *Environmental Toxicology and Pharmacology* **2014**, *37*, 1236–1242, doi:10.1016/j.etap.2014.04.020.
3. Li, T.; Dong, S.; Wang, E. A Lead(II)-Driven DNA Molecular Device for Turn-On Fluorescence Detection of Lead(II) Ion with High Selectivity and Sensitivity. *J. Am. Chem. Soc.* **2010**, *132*, 13156–13157, doi:10.1021/ja105849m.
4. Yu, Z.; Zhou, W.; Han, J.; Li, Y.; Fan, L.; Li, X. Na^{+} -Induced Conformational Change of Pb^{2+} -Stabilized G-Quadruplex and Its Influence on Pb^{2+} Detection. *Anal. Chem.* **2016**, *88*, 9375–9380, doi:10.1021/acs.analchem.6b02466.
5. Wu, Y.; Shi, Y.; Deng, S.; Wu, C.; Deng, R.; He, G.; Zhou, M.; Zhong, K.; Gao, H. Metal-Induced G-Quadruplex Polymorphism for Ratiometric and Label-Free Detection of Lead Pollution in Tea. *Food Chemistry* **2020**, 128425, doi:10.1016/j.foodchem.2020.128425.
6. Qian, Z.S.; Shan, X.Y.; Chai, L.J.; Chen, J.R.; Feng, H. A Fluorescent Nanosensor Based on Graphene Quantum Dots–Aptamer Probe and Graphene Oxide Platform for Detection of Lead (II) Ion. *Biosensors and Bioelectronics* **2015**, *68*, 225–231, doi:10.1016/j.bios.2014.12.057.
7. Geng, F.; Wang, D.; Feng, L.; Li, G.; Xu, M. An Improved Structure-Switch Aptamer-Based Fluorescent Pb^{2+} Biosensor Utilizing the Binding Induced Quenching of AMT to G-Quadruplex. *Chem. Commun.* **2020**, *56*, 10517–10520, doi:10.1039/D0CC03669B.
8. Liu, Y.; Guo, Q.; Qu, X.; Sun, Q. Supramolecularly Assembled Ratiometric Fluorescent Sensory Nanosystem for "Traffic Light"-Type Lead Ion or PH Sensing. *ACS Appl. Mater. Interfaces* **2018**, *10*, 30662–30669, doi:10.1021/acsami.8b10007.
9. Peng, D.; Li, Y.; Huang, Z.; Liang, R.-P.; Qiu, J.-D.; Liu, J. Efficient DNA-Catalyzed Porphyrin Metalation for Fluorescent Ratiometric Pb^{2+} Detection. *Anal. Chem.* **2019**, *91*, 11403–11408, doi:10.1021/acs.analchem.9b02759.
10. Xu, Y.; Meng, X.; Liu, J.; Dang, S.; Shi, L.; Sun, L. Luminescent Nanoprobes Based on Upconversion Nanoparticles and Single-Walled Carbon Nanohorns or Graphene Oxide for Detection of Pb^{2+} Ion. *CrystEngComm* **2016**, *18*, 4032–4037, doi:10.1039/C5CE02537K.
11. Chen, G.; Bai, W.; Jin, Y.; Zheng, J. Fluorescence and Electrochemical Assay for Bimodal Detection of Lead Ions Based on Metal–Organic Framework Nanosheets. *Talanta* **2021**, *232*, 122405, doi:10.1016/j.talanta.2021.122405.
12. Yu, L.; Zhao, H.; Jing, N.; Shi, X.; Zhang, Y.; Ding, C.; Zhang, Y.; Fan, J.; Tang, Y. A Supramolecular Probe of Cyanine Dye for Pb^{2+} Detection Based on the Recognition of a G-Quadruplex from DNA Duplexes. *Anal. Methods* **2020**, *12*, 1182–1185, doi:10.1039/C9AY02446H.
13. Khoshbin, Z.; Housaindokht, M.R.; Izadyar, M.; Bozorgmehr, M.R.; Verdian, A. Theoretical Design and Experimental Study of New Aptamers with the Improved Target-Affinity: New Insights into the Pb2+-Specific Aptamers as a Case Study. *Journal of Molecular Liquids* **2019**, *289*, 111159, doi:10.1016/j.molliq.2019.111159.
14. Song, H.-Y.; Kang, T.-F.; Jiang, M.-F.; Zhang, J.-J.; Cheng, S.-Y. A Novel Strategy Based on DNAzyme for Electrochemiluminescence Detection of Pb(II) with P-GO@QDs for Signal Amplification. *Journal of Electroanalytical Chemistry* **2017**, *801*, 244–250, doi:10.1016/j.jelechem.2017.07.051.
15. Xu, S.; Chen, X.; Peng, G.; Jiang, L.; Huang, H. An Electrochemical Biosensor for the Detection of Pb2+ Based on G-Quadruplex DNA and Gold Nanoparticles. *Anal Bioanal Chem* **2018**, *410*, 5879–5887, doi:10.1007/s00216-018-1204-6.
16. Ding, J.; Zhang, D.; Liu, Y.; Yu, M.; Zhan, X.; Zhang, D.; Zhou, P. An Electrochemical Aptasensor for Detection of Lead Ions Using a Screen-Printed Carbon Electrode Modified with Au/Polypyrrole Composites and Toluidine Blue. *Anal. Methods* **2019**, *11*, 4274–4279, doi:10.1039/C9AY01256G.
17. Xu, W.; Zhou, X.; Gao, J.; Xue, S.; Zhao, J. Label-Free and Enzyme-Free Strategy for Sensitive Electrochemical Lead Aptasensor by Using Metal-Organic Frameworks Loaded with AgPt Nanoparticles as Signal Probes and Electrocatalytic Enhancers. *Electrochimica Acta* **2017**, *251*, 25–31, doi:10.1016/j.electacta.2017.08.046.
18. Li, F.; Feng, Y.; Zhao, C.; Tang, B. Crystal Violet as a G-Quadruplex-Selective Probe for Sensitive Amperometric Sensing of Lead. *Chem. Commun.* **2011**, *47*, 11909, doi:10.1039/c1cc15023e.
19. Ebrahimi, M.; Raoof, J.B.; Ojani, R. Design of a Novel Electrochemical Biosensor Based on Intramolecular G-Quadruplex DNA

for Selective Determination of Lead(II) Ions. *Anal Bioanal Chem* **2017**, *409*, 4729–4739, doi:10.1007/s00216-017-0416-5.
